# Contribution and Future of High-Throughput Transcriptomics in Battling Tuberculosis

**DOI:** 10.3389/fmicb.2022.835620

**Published:** 2022-02-24

**Authors:** Amparo Martínez-Pérez, Olivia Estévez, África González-Fernández

**Affiliations:** ^1^Biomedical Research Center (CINBIO), Universidade de Vigo, Vigo, Spain; ^2^Hospital Álvaro Cunqueiro, Galicia Sur Health Research Institute (IIS-GS), Vigo, Spain

**Keywords:** transcriptomics, tuberculosis, mycobacteria, RNA-sequencing, microarray, immune response, drug resistance

## Abstract

While Tuberculosis (TB) infection remains a serious challenge worldwide, big data and “*omic*” approaches have greatly contributed to the understanding of the disease. Transcriptomics have been used to tackle a wide variety of queries including diagnosis, treatment evolution, latency and reactivation, novel target discovery, vaccine response or biomarkers of protection. Although a powerful tool, the elevated cost and difficulties in data interpretation may hinder transcriptomics complete potential. Technology evolution and collaborative efforts among multidisciplinary groups might be key in its exploitation. Here, we discuss the main fields explored in TB using transcriptomics, and identify the challenges that need to be addressed for a real implementation in TB diagnosis, prevention and therapy.

## Introduction

### The Challenge of Tuberculosis Disease

Tuberculosis (TB) remains a relentless disease caused by the bacillus *Mycobacterium tuberculosis*. With more than 1.5 million deaths annually, it is one of the top causes of death worldwide and the second leading cause from a single infectious agent, after COVID-19 (World Health Organization [WHO], 2021). Tuberculosis disease displays a continuous spectrum. After the bacteria enters the body, it can be rapidly eliminated by the innate immune system (“early clearance”) or it can persist, triggering the response of the adaptive immune system. Only 5–10% of the infected people directly progress to the active disease, whereas in most cases the mycobacteria enters into a dormant state and persists in the organism trough years, as latent TB infection (LTBI). This latent stage can eventually lead toward bacteria reactivation (“post-primary TB”) or the disease resolution.

LTBI has classically included those asymptomatic people that developed immune memory against TB. It is now stablished that other scenarios might occur, including rapid (“incipient disease,” with no evidence of active Mtb) or slow (“subclinical tuberculosis,” with radiological abnormalities or microbiological evidence of active Mtb) progression toward the active disease, cycling between stages, or the effective elimination of the infection after developing memory (Drain et al., 2018). Nonetheless, most routine diagnostic tests do not differentiate among these stages. It is estimated that about one-quarter of the world’s population has contacted with the bacilli and developed immune memory reflected in the definition of LTBI.

Understanding the factors that promote the diverse and dynamic stages of the infection, including early clearance of the pathogen and active disease, is therefore key to develop an appropriate strategy in the fight against TB.

The economic barriers between high and low-income areas usually affect TB diagnosis and treatment, as well as the outcome of the patient. Moreover, the increasing rates of multidrug-resistant (MDR-TB) and extensively drug-resistant TB (XDR-TB) threats TB management in the future. The failure in developing an effective vaccine for adult pulmonary TB, opens another front in TB research and in the control of the disease.

Development of high-throughput (HT) technologies were expected to impulse the understanding of the complexity of this disease. They enabled the revolution of the *omic* sciences, including genomics, transcriptomics, proteomics, epigenomics, or metabolomics.

The term transcriptomics refers to the study of the complete set of RNA transcripts produced by the genome at a specific time or circumstance. Their studies are being used in many areas of knowledge like toxicology, immunology, microbiology, environmental studies or evolutionary relationships among species. In medicine, it is a powerful tool for the understanding of many human diseases, including infections, cancer or autoimmune diseases.

Transcriptomics cannot predict the post-transcriptional protein modifications that usually affect the protein’s function. As a matter of fact, the comparison among transcriptome and proteome commonly fails to show a good correlation (Kumar et al., 2016). Nonetheless, the complexity of proteome analysis limits its understanding. Therefore, gene expression analysis are routinely accepted as a proxy for biological activity, being more affordable and manageable than proteomics. Furthermore, transcriptomics allows the detection of low abundant gene transcripts, and the identification of novel isoforms resulting from alternative splicing or single nucleotide polymorphisms, where proteomic fails. Researchers must understand and accept the limitations of both systems when choosing their approach.

Given these limitations, studies that include both approaches can provide a more complete picture of the processes that characterize the disease. A combination of *omic* approaches with immune profiling techniques, such as flow cytometry, *in vitro* cell activation or measurement of cytokine secretion, etc., can help to identify the immunological mechanisms involved in the different stages of the disease, which is key in the development of both diagnostic and treatment approaches.

### Transcriptome Studies: Exploring the Past…

Transcriptome can be partially studied using targeted expression approaches, as real-time quantitative reverse transcription PCR (RT-qPCR) or differential display for measuring gene expression, or RNA interference for exploring gene function. Nonetheless, the usage of high throughput technologies allows the unbiased acquisition of tens of thousands of potential candidate genes in just one step.

The first approaches for the gene expression analysis emerged in the early 1990 ìs and were based on Sanger sequencing. They included Expressed Sequence Tag (EST) libraries, Serial Analysis of Gene Expression (SAGE), Cap Analysis of Gene Expression (CAGE), and Massively Parallel Signature Sequencing (MPSS). They had various limitations, including high costs due to the Sanger sequencing technology, dependence on enzyme recognition sites, and limitations in detecting isoforms (Wang et al., 2009). Reverse-transcriptase multiplex ligation-dependent probe amplification (RT-MLPA) is also used to quantify expression of a predefined list of target genes, but fails in assessing hundreds of genes simultaneously (Eldering et al., 2003).

**FIGURE 1:**
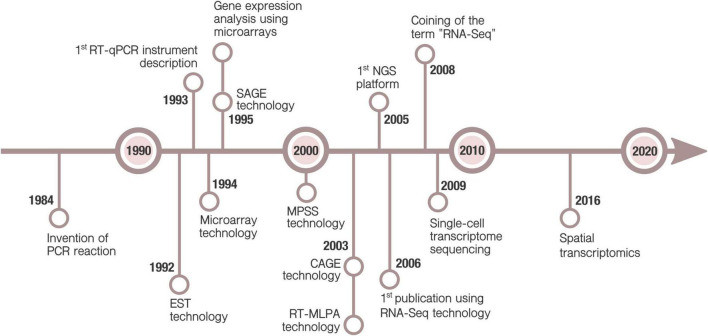
Timeline of main milestones in technologies for gene expression. Polymerase Chain Reaction (PCR) was invented in 1984 by Mullis (1990). A decade later, Real-Time qPCR (RT-qPCR) technology enabled the detection of PCR products in real-time (Higuchi et al., 1993). This allowed, for the first time, quantify mRNA expression of selected genes. Simultaneous analysis of multiple genes was possible due to microarray, Expressed Sequence Tag (EST) (Okubo et al., 1992), Serial Analysis of Gene Expression (SAGE) (Velculescu et al., 1995), Massively Parallel Signature Sequencing (MPSS) (Brenner et al., 2000), Cap Analysis of Gene Expression (CAGE) (Shiraki et al., 2003) and Reverse-transcriptase multiplex ligation-dependent probe amplification (RT-MLPA) (Eldering et al., 2003) technologies. Although the DNA hybridization method was described earlier, the microarray technology is considered to be firstly commercialized by Affymetrix in 1994 (Lenoir and Giannella, 2006). It was promptly applied to measure gene expression (Schena et al., 1995). It continues to be one of the most popular methods for gene expression, allowing the analysis of hundreds or thousands of genes. Development of Next Generation Sequencing (NGS) in the onset of 2000s supposed a revolution for both genomics and transcriptomics (Mardis, 2011). The first publication using NGS RNA-Seq technology was in 2006 (Bainbridge et al., 2006), but it was not until 2008 when the term RNA-seq started to be used (Lister et al., 2008; Mortazavi et al., 2008). RNA-Seq was applied to single-cell technology in 2009 for the first time (Tang et al., 2009). Emerging applications, as spatial transcriptomics (Ståhl et al., 2016), hold the promise of new advances in transcriptomics research.

These methods were soon replaced by hybridization-based microarray technology which has a much better affordability for large scale studies. Next-generation sequencing, and thus RNA-Seq, arose later, in mid 2000s (Figure 1). Currently, high-throughput transcriptomics (HTTr) rely on those two platforms.

For some time, RNA microarrays were nonetheless preferred over RNA-Seq because of its reduced price. Now, the equalization of costs of both techniques have favored the popularity of RNA-Seq for exploratory research, as which present various advantages over microarrays, but also have a few shortcomings (Table 1). In TB, where the discovery of novel biomarkers is still required, transcriptomics and RNA-Seq are considered the most informative and cost effective assays to start with. Once established a set of genes of interest, further exploration can be done using a well-designed microarray or by RT-qPCR, reducing complexity and costs.

**TABLE 1:** Advantages and disadvantages of microarrays and RNA-sequencing for gene expression analysis.

	Microarray	RNA-seq
Advantages	• Standardized analysis: Easier to analyze. • Moderately lower cost.	• Broader dynamic range (< 10^5^) and sensitivity (Zhao et al., 2014). • Allows modification of sequencing coverage depth to detect rare and low-abundance transcripts. • Allows the detection of novel transcripts, splicing variants, single nucleotide variants, insertions, deletions and gene fusions.
Disadvantages	• Lower dynamic range (10^3^–10^4^). • Does not allow the detection of novel transcripts (uses pre-designed probes).	• Large size of files: Demands considerable amount of computer resources for storage and analysis. • Lack of standardized protocol for analysis: Complex computational analysis and longer analysis times.

Another major contributor to the transcriptomics success is the development of single-cell RNA-Seq technology and spatial transcriptomics. While single-cell proteomics is still in its infancy, single-cell transcriptomics allows an unprecedent analysis of gene expression and its heterogeneity amongst different cell populations, rare cell detection or complex interactions among tissues (Cai et al., 2020; Nathan et al., 2021; Pisu et al., 2021). Some drawbacks are its elevated cost, with reagents 10–20 times more expensive than conventional RNA-Seq, and the complexity of its analysis, facing new computational challenges such as the necessity of dimensionality reduction and robust statistics to detect true heterogeneity (Adil et al., 2021). Spatial transcriptomics, which combines gene expression analysis and histology, allows for the first time the localization of mRNA molecules within a tissue or identifying their subcellular location (Ståhl et al., 2016). It has rapidly become a promising tool in research and diagnostics, with special applications in assessing tissue heterogeneity and cell interactions.

HTTr studies have boosted the amount of available information to levels never reached before. The large-scale data obtained requires of bioinformatics tools to be adequately processed and understood. Data can be stored in either private or public storage. Most scientific publications require the transcriptomic raw data to be available in public repositories, where it can be freely downloaded. Some examples of public repositories for transcriptomic data can be found in Table 2.

**TABLE 2:** Public repositories for transcriptomic data in health sciences.

Repository	Host institution	File storage	Data type
Gene Expression Omnibus (GEO)	National Center for Biotechnology Information (NCBI)	NCBI Sequence Read Archive (SRA)	Functional genomics data generated from microarray or NGS platforms
BioStudies (former ArrayExpress)	European Bioinformatics Institute (EMBL-EBI)	European Nucleotide Archive (ENA)	Functional genomics data generated from microarray or NGS platforms
DDBJ Sequence Read Archive (DRA)	DNA Data Bank of Japan (DDBJ)	DDBJ Sequence Read Archive (DRA)	Functional genomics data generated from NGS platforms.
Genomic Data Commons (GDC)	National Cancer Institute (NCI)	Genomic Data Commons (GDC)	Functional genomics data generated from NGS platforms in cancer.
Genome Sequence Archive (GSA)	National Genomics Data Center (NGDC), China National Center for Bioinformation (CNCB)	Genome Sequence Archive (GSA)	Raw sequence reads from diverse sequencing platforms

*NGS, Next-Generation Sequencing.*

Nonetheless, special care must be taken when comparing data from different sources. A meticulous annotation must be done when sharing our data, including the experimental protocol, procedure for sample isolation, relevant donor information, microarray design or RNA-Seq technology used or selection of either total RNA or mRNA fraction for RNA-Seq. The availability of a complete and curated metadata will ease further *in silico* analysis comparing data worldwide.

**FIGURE 2:**
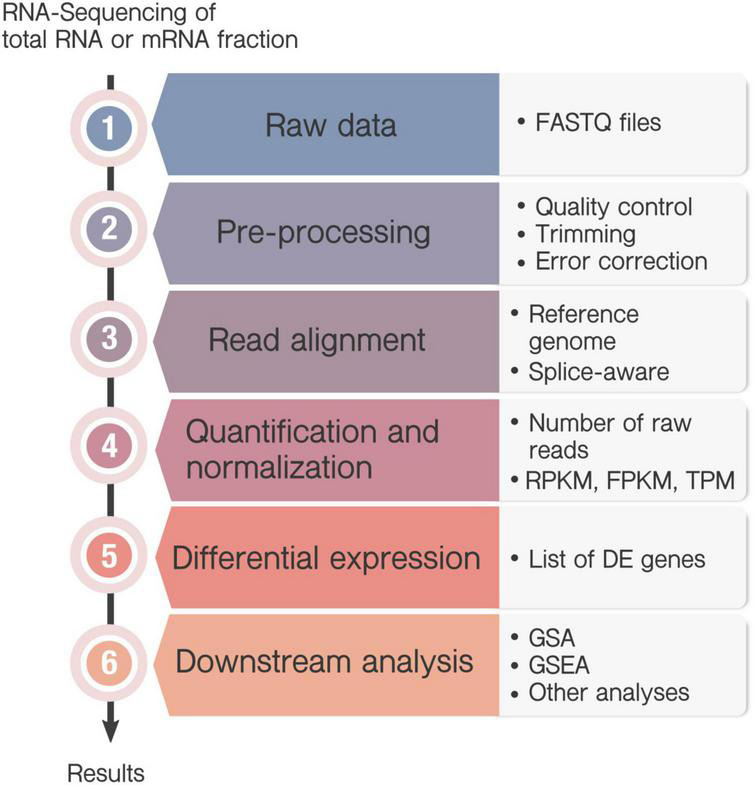
Workflow in RNA-Seq analysis. Transcriptome can be sequenced either from messenger RNA (mRNA) fraction, or total RNA, which includes also ribosomal RNA and transfer RNA. (1) RNA-sequencing generate a large amount of data from the millions of sequenced fragments (reads), and converts the information into a FASTQ file. (2) Pre-processing steps are commonly performed including quality check, trimming, filtering or error correction. (3) If an annotated genome is available, the sequenced reads are mapped onto the reference genome to identify each transcript and the correspondent gene. In this case, it is recommended to use splice-aware aligners, that align reads across splice junctions. However, if a reference genome is not available, then the reads will be assembled *de novo* by their overlapping regions to form contigs. (4) Next, quantification determines the number of raw reads that map to each transcript or gene and commonly normalized them to be compared between samples. The most commonly used normalizations are the “Reads Per Kilobase Million” (RPKM) or its alternative “Fragments Per Kilobase Million” (FPKM) and the “Transcripts per Kilobase” (TPM). (5) Then, differential expression (DE) analysis allows the identification of those genes whose expression change under particular circumstances indicates the gene expression profile associated to a certain condition through different statistical methods. (6) The result of a differential expression analysis is a list of DE genes that can sometimes contain hundreds or even thousands of genes. A downstream analysis is usually needed to interpret the results, as Gene Set Analysis (GSA) or Gene Set Enrichment Analysis (GSEA). Besides, there are many other options for the analysis of RNA-seq data, as the identification of Single Nucleotide Polymorphisms, or nucleotide insertions and deletions.

An increasing number of bioinformatics tools are developed for the different steps of the transcriptome analysis, with many alternatives available for each step. RNA-Seq analysis share some common steps (Figure 2), but there is not a unique pipeline that one can follow and the pipeline of choice depends mainly on the biological question of interest. To take full advantage of our data, it is desired to keep updating our knowledge of useful and reliable software. The sustained software development also hinders experts in the field to review and criticize how most data is analyzed. Lastly, communication issues among biological scientists and data analysts may also become a handicap to get the most out of the experimental data.

## Transcriptomics Applied to Tuberculosis Research

It has been two decades since the first HTTr studies in TB were published (Ehrt et al., 2001; Ragno et al., 2001). HTTr have been used to explore different features, of both host and bacteria. Work is commonly not limited to Mtb but it includes the *Mycobacterium tuberculosis complex* (MTBC) species, the genetically related etiologic agents of tuberculosis in humans or other animals.

In this review, we will navigate through some of the most relevant HTTr studies performed in TB to date. We will not delve into the posterior evaluation and validation of the postulated biomarkers. Studies have been classified in sections according to their contributions, although they usually served to more than one purpose. A summary of applications and references can be found in Table 3.

**TABLE 3:** Summary of high-throughput transcriptomic applications in TB research.

Field in TB research	Applications	References
Mechanisms of bacterial infection	• Characterize Mtb growing *in vitro*.	Fisher et al., 2002; Stewart et al., 2002; Ohno et al., 2003; Bacon et al., 2004; Rustad et al., 2008; Mehra and Kaushal, 2009; Voskuil et al., 2011; Tan et al., 2013; Lin et al., 2016; Saini et al., 2020
	• Characterize mutant isogenic strains and clinical isolates.	Manganelli et al., 2002; Stewart et al., 2002; Gao et al., 2005; Rose et al., 2013; Eldholm et al., 2014; Gomez-Gonzalez et al., 2019; Saini et al., 2020
	• Characterize Mtb infecting cells *in vitro*.	Chaussabel et al., 2003; Schnappinger et al., 2003; Cappelli et al., 2006; Rachman et al., 2006a; Rohde et al., 2007, 2012; Fontán et al., 2008a,b; Zimmermann et al., 2017
	• Gene expression changes during *in vivo* infection.	Talaat et al., 2004; Rengarajan et al., 2005; Rachman et al., 2006b; Gautam et al., 2015; Coppola et al., 2016; Sharma et al., 2017; Pisu et al., 2020, 2021
	• Biofilm production.	Flores-Valdez et al., 2020
Mechanisms of latency	• Model of latency *in vitro*.	Sherman et al., 2001; Betts et al., 2002; Park et al., 2003; Bacon et al., 2004; Hampshire et al., 2004; Muttucumaru et al., 2004; Voskuil et al., 2004; Beste et al., 2007; Murphy and Brown, 2007; Balázsi et al., 2008; Deb et al., 2009; McGillivray et al., 2015; Aguilar-Ayala et al., 2017
Mechanisms of host response	• Characterize the host immune response to infection, analyzing blood or lung tissue.	Berry et al., 2010; Jacobsen et al., 2010; Kim et al., 2010; Maertzdorf et al., 2011a; Ottenhoff et al., 2012; Joosten et al., 2013; Arlehamn et al., 2014; Singhania et al., 2018; Cai et al., 2020; Weiner et al., 2020
	• Understand the bases of early clearance.	Weiner et al., 2020
	• Function of host non-coding regulatory RNA.	Liu et al., 2011; Sharbati et al., 2011; Wang et al., 2011
	• Dual transcriptomic analysis to comprehend host-bacteria interaction.	Fontán et al., 2008a; Rienksma et al., 2015; Zimmermann et al., 2017; Pisu et al., 2020, 2021
Diagnosis	• Identify blood biomarkers that differentiate active, latent TB patients or healthy individuals.	Jacobsen et al., 2007; Mistry et al., 2007; Berry et al., 2010; Maertzdorf et al., 2011a,b; Anderson et al., 2014; Cai et al., 2014; Zak et al., 2016; Singhania et al., 2018; Estévez et al., 2020
	• Biomarkers that differentiate TB from other infectious diseases.	Berry et al., 2010; Maertzdorf et al., 2012; Bloom et al., 2013; Kaforou et al., 2013; Anderson et al., 2014; Hoang et al., 2021
	• Biomarkers for extrapulmonary TB.	Roe et al., 2016
	• Biomarkers for HIV-TB coinfection.	Kaforou et al., 2013; Anderson et al., 2014
Treatment evolution	• Identify biomarkers of success/failure to anti-TB treatment.	Berry et al., 2010; Bloom et al., 2012; Ottenhoff et al., 2012; Cliff et al., 2013, 2016; Zak et al., 2016; Thompson et al., 2017; Penn-Nicholson et al., 2020; Tabone et al., 2021
Progression to TBI	• Finding biomarkers that predict progression to active TBI.	Maertzdorf et al., 2011b; Zak et al., 2016; Scriba et al., 2017; Suliman et al., 2018; Warsinske et al., 2019; Ahmed et al., 2020; Estévez et al., 2020; Gupta et al., 2020; Moreira-Teixeira et al., 2020b; Roe et al., 2020; Burel et al., 2021; Esaulova et al., 2021; Nathan et al., 2021; Tabone et al., 2021
	• Characterize patients at risk of recurrent TB.	Mistry et al., 2007
Drug resistance and search for novel drugs	• Understand mechanisms underlying Mtb single and multi-drug resistance *in vitro*.	Keren et al., 2011; Chatterjee et al., 2013; Knegt et al., 2013; Peñuelas-Urquides et al., 2013; Eldholm et al., 2014; Blondiaux et al., 2017; Zheng et al., 2018
	• Identify bacterial candidates for drug targeting.	Betts et al., 2002; Talaat et al., 2004; Gao et al., 2005; Cappelli et al., 2006; Rachman et al., 2006b; Murphy and Brown, 2007; Balázsi et al., 2008; Fontán et al., 2008a; Rustad et al., 2008; Deb et al., 2009; Salamon et al., 2014; Defelipe et al., 2016; Aguilar-Ayala et al., 2017; Sharma et al., 2017; Banerjee et al., 2020; Saini et al., 2020
	• Unravel the mechanism of action of novel compounds.	Boshoff et al., 2004; Waddell et al., 2004; Manjunatha et al., 2009; Foo et al., 2018; Zheng et al., 2018
Vaccines and correlates of protection	• BCG vaccination effect	Fletcher et al., 2009, 2016; Arts et al., 2018; Cirovic et al., 2020
	• Characterization of diverse BCG strains and effect in vaccination.	Behr et al., 1999; Brosch et al., 2007; Abdallah et al., 2015
	• Profile immune response generated by novel TB vaccine candidates.	Zárate-Bladés et al., 2009; Gengenbacher et al., 2016; Hoft et al., 2016; Loxton et al., 2017; Hansen et al., 2018; Santoro et al., 2018; van den Berg et al., 2018; Martínez-Pérez et al., 2021
	• Search for correlates of protection for new vaccines design or therapies.	Aranday Cortes et al., 2010; Maertzdorf et al., 2011b; Fletcher et al., 2016; Hansen et al., 2018; Weiner et al., 2020; Martínez-Pérez et al., 2021

*BCG, Bacillus Calmette-Guérin; HIV, Human Immunodeficiency Virus; Mtb, Mycobacterium tuberculosis; MTBC, Mycobacterium Tuberculosis Complex; TB, Tuberculosis.*

### Mechanisms of Tuberculosis Infection

TB HTTr studies can be addressed from two different perspectives: (i) one focused on the mechanisms used by the mycobacteria to infect the host and escape the immune system, or (ii) on the host’s immune defense itself.

Focusing on the mycobacteria mechanisms of infection, Mtb is able to adapt to different scenarios through the course of infection, changing its gene expression accordingly. The characterization of the bacteria response in different microenvironment will improve our understanding of TB pathogenesis and how it escapes from the immune response.

*In vitro* culture is one of the easiest way to study the mycobacteria. Microarray and RNA-Seq have been used to study its growth in broth culture (either exponential or stationary phase), or after being exposed to a variety of perturbations that mimic the infection process. Low pH (Fisher et al., 2002; Tan et al., 2013), low oxygen (Bacon et al., 2004; Rustad et al., 2008), oxidative stress (Ohno et al., 2003; Mehra and Kaushal, 2009; Voskuil et al., 2011) or other perturbations (Stewart et al., 2002; Tan et al., 2013; Lin et al., 2016; Saini et al., 2020) are commonly used to recreate the intra-macrophage phagosomal environment. These studies have shown that environmental stress triggers different survival mechanisms in Mtb. For instance, the two-component regulator phoPR in response to Cl- in synergy with pH (Tan et al., 2013); genes involved in the non-ribosomal-peptide synthesis and fatty acid metabolism in low-pH environment (Fisher et al., 2002); the biosynthesis of cell wall precursors in response to low oxygen (Bacon et al., 2004), sigma factors (Mehra and Kaushal, 2009) or gene expression regulation induced by reactive nitrogen species, via overlapping signal transduction pathways (Ohno et al., 2003).

Diversity of MTBC members are used: wild-type common research strains (Abdallah et al., 2015), mutant strains (Manganelli et al., 2002; Stewart et al., 2002; Saini et al., 2020), or clinical isolates to characterize them (Gao et al., 2005; Rose et al., 2013; Eldholm et al., 2014; Gomez-Gonzalez et al., 2019). A great part of the work done with mutants use genetically modified isogenic strains, constructed to assess the function of a certain gene. This input has been key to comprehend most bacterial virulence factors, including the *acr-2* gene, one of the most prominent features of the Mtb heat-shock response (Stewart et al., 2002).

However, *in vitro* bacterial growth cannot completely simulate the host intracellular environment. Capturing the transcriptome of mycobacteria inside macrophages or dendritic cells is technically difficult because of the low abundance and quality of bacterial RNAs (Hinton et al., 2004) and hence there are limited data available. Pioneer work by Schnappinger et al. (2003) captured for the first time the intra-phagosomal Mtb transcriptome. They infected bone-marrow macrophages from wild-type and mutant mice and profiled the Mtb transcriptome in different conditions. Data revealed how Mtb upregulate genes in the phagosome required for fatty acid degradation, siderophore synthesis, DNA repair, cell envelope remodeling, lead to anaerobic respiration and increase iron uptake. Other studies followed their lead, including *in vitro* infection of human (Chaussabel et al., 2003; Cappelli et al., 2006; Fontán et al., 2008a,b; Zimmermann et al., 2017) or mouse macrophages (Rachman et al., 2006a; Rohde et al., 2007, 2012).

It is of special interest to define the bacterial expression changes occurring inside pulmonary tissue, the main site of infection, and especially from human TB samples. Nevertheless, *in vivo* infection has been mostly studied using animal models (Talaat et al., 2004; Rengarajan et al., 2005; Gautam et al., 2015; Coppola et al., 2016; Pisu et al., 2020, 2021) and have described how the bacteria changes its transcriptome within days of infection (Talaat et al., 2004; Coppola et al., 2016). Only a few articles have worked directly with samples from infected TB patients (Rachman et al., 2006b; Sharma et al., 2017). Another area explored by HTTr is the biofilm production by *Mycobacterium* species (Flores-Valdez et al., 2020).

Mycobacterial features drawn from these studies could also be used as targets for novel TB drugs and therapies. However, further research is required due to the plasticity of the response depending on the infection status, the microenvironment and the differences in methodology among publications.

### Mechanisms of Mtb Latency

One of the most remarkable Mtb features is its ability to adapt to stress conditions generated by the host by entering into latency. This dormant phase is characterized by growth arrest, anaerobic respiration and antibiotic tolerance. Understanding the mechanisms behind the latent phase and how it is switched would help battling persistent TB. In physiological conditions, latent bacteria will be mostly contained into granulomas, multicellular structures comprised of macrophages, T cells, other cell types and biomolecules.

*In vitro* studies have tried to recreate the conditions believed to occur inside the granuloma. In order to do that, HTTr have been applied to MTBC cultures in hypoxia (Sherman et al., 2001; Bacon et al., 2004; Muttucumaru et al., 2004; Voskuil et al., 2004; McGillivray et al., 2015), starvation (Betts et al., 2002; Hampshire et al., 2004) or a combination of conditions (Beste et al., 2007; Deb et al., 2009; Aguilar-Ayala et al., 2017).

Isogenic mutant strains were tested in dormancy-like conditions to understand the role of diverse genes in latency (Park et al., 2003; McGillivray et al., 2015). Some key genes involved in transition to latency were discovered trough these HTTr studies, as the DosR regulon (Park et al., 2003). A meta-analysis of Mtb under latency-like conditions analyzed sets of genes regulated by transcription factors, and found differences during its adaptation (Balázsi et al., 2008). They agreed that dosR-controlled genes were the most responsive early during infection, while nadR, sigE, sigC, and furB regulons peaked later, after day 20. Other meta-analysis searched promising latency targets for drug discovery (Murphy and Brown, 2007).

Study of latent mycobacteria *in vivo* gets more problematic. The most widely used laboratory mouse strains do not generate caseous granulomas. As a consequence, specific mice strain (C3HeB/FeJ) or other animals as guinea pigs are commonly used, raising the experimental costs. Regarding human studies, obtaining granulomas from living patients requires delicate surgery, which is only recommended for patients with severe lung disease. To the best of our knowledge, transcriptome data from latent MTBC bacilli *in vivo* are not publicly available yet. Exploring TB latency remains technically challenging, but warrant further investigation.

### Mechanisms of Host Immune Response

Along with the mycobacteria, the host response can swing the outcome of the disease. There are many unsolved questions about how some individuals develop the active disease while others go through latent infection or are able to clear the mycobacteria early.

Host transcriptional studies in human using easily collectible samples, like blood or saliva, had helped both exploring the immunopathogenesis of TB and identifying diagnostic markers. A pioneer work was conducted by Berry et al. (2010), demonstrating that blood transcriptome could reflect part of the events taking place at the site of infection. Such study provided a 393-gene signature associated with active TB able to discriminate between active and LTBI.

Following their steps, posterior studies have searched for specific features of active and/or latent TB disease (Berry et al., 2010; Maertzdorf et al., 2011a; Ottenhoff et al., 2012; Singhania et al., 2018) that have provided a greater insight into the host immune mechanisms that take place during the infection. Crucial TB biomarkers were derived from these works, including the first evidence of the importance of type I interferon in the pathogenesis of human tuberculosis (Berry et al., 2010); alteration of pathways related to regulation of immune responses; signal transduction and activation of leukocyte populations; intracellular protein trafficking; cell structure and motility changes; cell cycle control, phagocytosis, endocytosis and activation of the complement cascade. The diagnostic applications of these and other studies are further detailed in the TB diagnosis section.

Integration of results from independent studies can offer a new vision, such as the suggestion of a strong dominant role for myeloid cells in TB inflammation (Joosten et al., 2013). Additionally, application of single-cell transcriptomics enabled the identification of novel biomarkers and cell populations participating in TB previously undescribed, such as the depletion of the natural killer cell subset CD3^–^CD7^+^GMZMB^+^in TB (Cai et al., 2020).

Further efforts profiled the host response by isolating certain cell subtypes from blood and analyzing their specific transcriptional response (Jacobsen et al., 2010; Arlehamn et al., 2014). The bases of early clearance have also been investigated, being able to predict conversion to tuberculin skin test positive of household contacts, prior to any signs of infection (Weiner et al., 2020). Nonetheless, blood profiling may miss factors of biological relevance only present in the real environment of infection.

Transcriptomic studies using cells from the site of infection from real TB patients remain scarce, mainly owing to the difficulty of obtaining samples. Kim et al. (2010) successfully isolated caseous granulomas from TB patients, finding abundant transcripts involved in various destructive tissue pathologies and upregulated genes for lipid sequestration and metabolism. Importantly, the granuloma transcriptome did not entirely correlate with published TB blood signatures. Despite the difficulty, a proteomic human granuloma signature has also been accomplished (Marakalala et al., 2016). Future work deciphering the granuloma environment will have to deal with the enormous heterogeneity of granulomas within the host. Other approaches have captured the transcriptional response of cells after *in vitro* Mtb infection, using either human (Chaussabel et al., 2003; Volpe et al., 2006; Fontán et al., 2008a; Salamon et al., 2014; Zimmermann et al., 2017) or animal cells (Ehrt et al., 2001; Schnappinger et al., 2003; Shi et al., 2003; Roy et al., 2018).

In these settings, *in vivo* infection in animal models are crucial. They allow not only to harvest a higher amount of sample than in patients, but also grant control and decision over infection conditions. Transcriptome analysis has been commonly done in blood (Moreira-Teixeira et al., 2020a,b) or lungs (Seiler et al., 2003; Mehra et al., 2010, 2015; Huang et al., 2018; Ahmed et al., 2020; Moreira-Teixeira et al., 2020b; Pisu et al., 2020) from mice or macaques. Some of these works beautifully isolated alveolar (AM) and interstitial macrophages (IM) from lung, obtaining their unique signatures after infection (Huang et al., 2018; Pisu et al., 2020). Other projects made progress toward the correlation of the TB signature among human and animal models. Ahmed et al. (2020) identified common transcriptional immune correlates of TB across lung from animal models and human blood. Moreira-Teixeira et al. (2020b) identified a blood signature in TB-susceptible C3HeB/FeJ mice infected with Mtb HN878 that is compatible with the human signature, whereas it is minimally altered in blood from TB-resistant C57BL/6J mice infected with Mtb H37Rv.

Again, single-cell RNA-Seq technology also provided novel insights in the field. Pisu et al. (2021) described the functional heterogeneity in AM and IM populations in mouse infected lungs. An outstanding work by Khan et al. (2020) using bulk and single-cell RNA-seq, demonstrated that Mtb is able to reprogram hematopoietic stem cells in the bone marrow, limiting myelopoiesis and impairing trained macrophage immunity. HTTr can also be used to analyze changes in the microRNA fraction (Liu et al., 2011; Sharbati et al., 2011; Wang et al., 2011) during mycobacteria infections.

Lastly, the usage of dual transcriptomic analysis, analyzing in parallel both the host and the bacteria transcriptomes, is an interesting approach with growing presence (Fontán et al., 2008a; Rienksma et al., 2015; Zimmermann et al., 2017; Pisu et al., 2020, 2021). This method provides a complete vision of how both organisms interact within each other at a precise time. For instance, it was found a link between bacterial stress, induction of drug tolerance and the expression levels of CD11c in macrophages (Pisu et al., 2021). In other work, Mtb growing inside AMs (their favorite niche) were reported to upregulate genes of oxidative phosphorylation, mitochondrial function, iron storage, cell division and growth (Pisu et al., 2020). Contrarily, Mtb inside IMs expressed more genes of bacterial stress, concurring with the upregulation of the host pro-inflammatory response by those macrophages. Other studies linked the bacterial SigE regulon with the modulation of the inflammatory response (Fontán et al., 2008a); or decipher the pathogen’s diet inside the macrophages (Zimmermann et al., 2017) by combining dual sequencing and metabolomics.

### Tuberculosis Diagnosis

TB diagnosis remain challenging. The current available tests present diverse shortcomings; i.e., microscopy has poor sensibility with low bacteria abundance; culture of Mtb requires weeks to grow; *ex vivo* Mtb gene amplification test Xpert MTB/RIF is expensive and fails to discriminate cleared infections; etc. Besides, it is common to mislead TB with other diseases due to its variable clinical signs. An accurate diagnosis would translate into a more effective use of drug resources and to the identification of individuals that will benefit from preventive treatment. The advances in transcriptomic studies hold the promise of finding new biomarkers that will serve to accurately diagnose the different stages of TB infection.

The first ever published HTTr work in human TB diagnostic was performed by Mistry et al. (2007). They compared whole-blood from a reduced number of patients with active tuberculosis, latently infected, cured TB or recurrent TB. Ensuing works followed their lead, while the technological progress allowed a better characterization of the disease, with higher number of target genes and larger number of samples.

Although slight differences can be found in several studies, the majority characterizes the TB response comparing between active TB patients (TBI) with uninfected individuals (NoTBI) and latent healthy patients (LTBI) (Jacobsen et al., 2007; Berry et al., 2010; Maertzdorf et al., 2011a,b; Anderson et al., 2014; Cai et al., 2014; Singhania et al., 2018; Estévez et al., 2020). In most published studies, the differential expression of a group of genes is usually sufficient to classify these groups. Some highlighted genes or pathways include an interferon-inducible neutrophil-driven gene signature (Berry et al., 2010), lactoferrin, CD64, Ras-associated GTPase 33A (Jacobsen et al., 2007), changes in FCGR1B signaling (Maertzdorf et al., 2011b), or complement pathways (Cai et al., 2014). MicroRNAs (Fu et al., 2011; Zhang et al., 2013) have also been screened for TB diagnostic purposes.

The scientific community has made efforts in discriminating the TB gene signature from extrapulmonary TB (Roe et al., 2016) and other diseases (Berry et al., 2010; Maertzdorf et al., 2012; Bloom et al., 2013), specially other pulmonary diseases or co-infecting diseases that are highly prevalent in TB endemic areas. Multicenter studies (Kaforou et al., 2013; Anderson et al., 2014; Hoang et al., 2021) included a superior number of patients suffering from TB and other diseases, including HIV-coinfection.

Despite efforts, human TB biomarkers have shown little overlap between studies. An endless variety of factors may contribute to the signature diversity: differences in cohorts, variations in study design, diagnostic method for confirming TB, misclassified or undetected pathologies, missing data, BCG vaccination, transcriptomics platforms or analysis pipeline. Metadata analysis have exploited available data sets in an attempt to conceal existing studies (Sweeney et al., 2016; Warsinske et al., 2019; Gupta et al., 2020). A three-gene set (*GBP5, DUSP3*, and *KLF2*) defined by Sweeney et al. (2016), ascertain later in an independent study (Warsinske et al., 2019), was robust enough to diagnose active tuberculosis among their data.

Nonetheless, there is still a need for TB diagnostic biomarkers to be used in a heterogeneous population. Most successful studies were based on cohorts of hundreds of cases, with standardized sample processing methods and correct identification of the patients. The use of a validation cohort from another ethnicity and TB endemicity seems to be key to test the robustness of the transcriptomic signature. It is also highly desirable to compare to diseases closely related to TB in order to ensure the specificity of the infection. Moreover, the usage of samples different from blood, such as sputum or bronchoalveolar lavage, could reflect better what is happening at the site of disease, as they might expose useful biomarkers that are not so evident in blood. However, the difficulty in obtaining those samples and its limited reproducibility are major drawbacks.

### Biomarkers of Treatment Evolution

HTTr have also been applied to screen blood biomarkers that correlate with the response to anti-TB treatment. Those biomarkers would permit a rapid assessment of the treatment’s success or failure and detect patients that require a change in the treatment regimen. Implementation of the treatment monitoring in clinical practice will have a beneficial impact on patient wellness and health care costs.

Changes in the blood transcriptome of TB patients have been detected during different stages of the treatment or after treatment completion (Berry et al., 2010; Bloom et al., 2012; Ottenhoff et al., 2012; Cliff et al., 2013; Tabone et al., 2021). Generally, authors found a down-regulation of immune or inflammation-related genes coinciding with the disease resolution. A set of differentially expressed genes previously identified as TB progression biomarkers (Zak et al., 2016) also proved to be successful in treatment monitoring (Penn-Nicholson et al., 2020). Furthermore, an appealing study highlighted a signature of treatment failure before treatment initiation (Thompson et al., 2017), while other work presented a signature for detecting relapse after successful treatment (Cliff et al., 2016).

### Progression to Tuberculosis Infection

Although there are many evidences that suggest a continuous spectrum of TB infection (Drain et al., 2018), its diagnosis is currently divided into two different stages: latent or active TB. A major drawback of the fight against TB is the inability to differentiate between latently infected patients that are at a higher risk of transitioning to an active disease.

Many efforts have been made into understanding and predicting progression to active TBI, both amongst latently infected population (Maertzdorf et al., 2011b; Zak et al., 2016; Scriba et al., 2017; Burel et al., 2021), contacts of TB patients (Suliman et al., 2018; Estévez et al., 2020; Roe et al., 2020; Nathan et al., 2021), or combined cohorts in a meta-analysis of published data (Warsinske et al., 2019; Gupta et al., 2020; Moreira-Teixeira et al., 2020b). These studies differentiate TBI progressors and non-progressors. Some results suggest that people at risk of progression show an inflammatory profile that include Type I/II interferon and complement genes in early stages before progression to active TB, and a higher expression of lymphoid, monocyte and neutrophil genes proximal to the disease onset (Scriba et al., 2017).

One of the most cited works identified and validated a 16 gene-signature that differentiate people at risk of developing the active disease (Zak et al., 2016). Warsinske et al. (2019) compared 16 published gene signatures and assessed that the three-gene set selected by Sweeney et al. (2016) for TB diagnosis also served to accurately identify individuals at high risk of progression. Comparison of TB risk across species showed that the mentioned 16-gene signature is enriched in progressors across animal models, and specified the genes that mediate protective (as *Stat1* and *Tap1*) or detrimental (*Batf2*, *Fcgr1*, and *Scarf1*) responses in mouse model (Ahmed et al., 2020). The study of Esaulova et al. (2021) used single-cell RNA-seq and CyTOF technology to study TB in macaques. They reported an augment of CD27^+^ NK cells in lungs during latency Mtb-control, whereas progression was characterized by plasmacytoid dendritic cells and T cells producing IFN responses. Our group applied a machine-learning prediction model to identify a subset of LTBI contacts whose transcriptional profile are suggestive of having a higher probability of developing active TB (Estévez et al., 2020). Tabone et al. (2021) have recently published a unique study of blood signatures across the TB spectrum, including incipient, subclinical, and active TB. Changes in blood gene expression were minimal in incipient TB, increased in patients with subclinical TB and became maximal when clinical TB was diagnosed. They also assessed the effect of anti-TB treatment, obtaining differential responses among the defined TB subgroups. Other related studies included the characterization of patients at risk for recurrent tuberculosis (Mistry et al., 2007).

Predicting potential progression to active TB using biomarkers could be usefully translated into clinical care, by treating latent infected cases prior to develop the disease or augmenting medical monitoring.

### Drug Resistance and Search for Novel Drugs

The emergence of MDR and XDR Mtb strains severely compromises TB treatment, and urges the discovery of new therapeutic anti-mycobacterial compounds. We previously assessed how comprehension of the bacterial transcriptome helped in identifying potential drug candidates against both active and dormant tuberculosis. Comparative analysis of Mtb transcriptome treated with different drugs has also been used to elucidate the mode of action of uncharacterized anti-mycobacterial compounds, to identify new mycobacterial targets, or to predict effective drug combinations. Besides, gene expression comparison of resistant vs. sensitive strains can be used to discern mechanisms of antibiotic resistance.

The transcriptome response of sensitive mycobacteria strains to diverse drugs has been explored, including isoniazid (Wilson et al., 1999; Betts et al., 2003; Boshoff et al., 2004; Waddell et al., 2004), rifampicin (Boshoff et al., 2004), ciprofloxacin (Sullivan et al., 2008), capreomycin (Fu and Shinnick, 2007), or vancomycin (Provvedi et al., 2009). It has also been applied to unravel the mechanisms of novel compounds that demonstrated good antimycobacterial activity *in vitro* (Manjunatha et al., 2009; Foo et al., 2018).

Some mechanisms of *Mycobacterium* antibiotic resistance are already known, while others remain undiscovered. To inspect further into those tolerance mechanisms, the transcriptome response of mutant-resistant and sensitive-wild type Mtb strains were compared in diverse studies. They included single-drug (Knegt et al., 2013; Zheng et al., 2018), or multiple drug resistance (Keren et al., 2011; Chatterjee et al., 2013; Peñuelas-Urquides et al., 2013; Eldholm et al., 2014). Some of the main findings of these studies included a possible role of genes *Rv0559c* and *Rv0560c* (Knegt et al., 2013) in rifampicine resistance; they also found a set of genes upregulated in Mtb persisters, including *UspA*, *SigF*, or *dnaE2* (Keren et al., 2011); described the downregulation of diverse genes in multidrug resistant isolates (Chatterjee et al., 2013), and the differential expression drug efflux and mycolic acid synthesis pathways (Eldholm et al., 2014). One work assessed the transcriptional changes triggered by SMARt-420, a molecule that reverses ethionamide-acquired resistance in Mtb (Blondiaux et al., 2017).

The majority of published studies were performed under *in vitro* laboratory growth condition. Few works have explored the transcriptional response of Mtb from clinical samples or *in vivo* models during treatment (Karakousis et al., 2008; Shaikh et al., 2021). Furthermore, latent bacteria are largely tolerant to antibiotics, mostly because common antibiotics target proteins expressed in replicating bacilli. It has been described an altered transcriptional response to antibiotics in the dormant stage (Karakousis et al., 2008; Keren et al., 2011). In this setting, Mtb genes expressed during latency are obvious candidates for drug targeting. Various studies addressed this issue (Defelipe et al., 2016; Aguilar-Ayala et al., 2017; Banerjee et al., 2020) and identified potential candidates, as phosphate synthase I3PS or specific proteins Rv1994c, Rv2780, and Rv3515c. However, when drawing conclusions, we must bear in mind that most data are collected during Mtb transition into dormancy, rather than during the rigorous latency stage.

In sum, discoveries in the field have helped to predict the mechanism of action of previously uncharacterized compounds, identifying potential drugs, novel drug targets, or understanding resistance. One of the difficulties of the analysis remains in differentiating the expression changes directly corresponding to the drug target, from those derived, including compensatory effects.

### Immune Response to Novel Vaccines and Correlates of Protection

The vaccine containing the Bacille Calmette-Gueìrin (BCG) is currently the only approved TB vaccine, but it shows a highly variable efficacy against pulmonary TB in adults. It consists in a live-attenuated form of *Mycobacterium bovis*. As the classical dogma of TB protection, attributed to interferon-γ and CD4 T cells, has partially failed (Kagina et al., 2010), new reliable biomarkers of protection are searched to direct the development of novel vaccines or therapies for TB.

Gene expression profiling of blood from BCG-vaccinated donors revealed interesting findings, as a decrease in the expression of cell adhesion molecules (Fletcher et al., 2009) or the upregulation of several immune-related genes (Fletcher et al., 2016). Currently, it is highly accepted that BCG triggers epigenetic changes in the immune cells, inducing trained innate memory (Kaufmann et al., 2018). A couple of publications have addressed the changes in both the transcriptome and the epigenome induced by the vaccine in humans (Arts et al., 2018; Cirovic et al., 2020).

Pulmonary transcriptome after BCG vaccination has been analyzed in various animal models, mostly in mice and macaques (Mollenkopf et al., 2006; Aranday Cortes et al., 2010; Mehra et al., 2013; Kunnath-Velayudhan et al., 2017). A singular study analyzing the granuloma lesions in BCG-vaccinated macaques found the upregulation of various cytokines and chemokines in the vaccinated individuals (Mehra et al., 2013).

Importantly, distinct BCG strains may generate different transcriptomic profiles in the host, which seems in line with the high heterogeneity of the BCG vaccine efficacy. Numerous publications focused on comparing the bacterial transcriptomes of the diverse BCG variants in order to understand their differences (Behr et al., 1999; Brosch et al., 2007; Abdallah et al., 2015).

Novel vaccine candidates are continuously being developed to substitute or boost BCG, and commonly the protective results are compared to BCG itself. A common approach has been developing mutants of BCG, which also helps in the understanding of the mechanisms of BCG functioning. Both the BCG bacterial transcriptome (Flores-Valdez et al., 2018) or the host transcriptional response (Gengenbacher et al., 2016; Hoft et al., 2016) are studied to characterize those new BCG-based vaccines. Other novel vaccine strategies included peptide/protein-based (Santoro et al., 2018; Martínez-Pérez et al., 2021), viral vectors (Hansen et al., 2018) or DNA vaccines (Zárate-Bladés et al., 2009). The transcriptome analysis usually find a reduced TB disease-associated signature, upregulation of antigen presenting pathways, and suggest potential biomarkers of protection.

The human transcriptomic profile to novel TB vaccines is scarce. Valuable lessons may be drawn from the most advanced candidates: M72/AS01_E_ (van den Berg et al., 2018) and VPM1002 (Loxton et al., 2017).

Discoveries of protective mechanisms can also be drawn from therapeutic or early protection studies, discussed in previous sections. Host-directed therapies is a new and emerging concept in the treatment of TB, where the host response can be boosted using diverse biological compounds, with or without adjunct antibiotics.

Although HTTr studies can prioritize some candidate biomarkers, further testing must be done to validate their true effectiveness. Powerful correlates of TB protection will accelerate the progress of new TB vaccine development and therapies.

## Discussion

### To Shape the Future

The future of transcriptomics seems bright. Comparing with what has happened with genome sequencing, we will expect that new equipment, cheaper reagents, specialized groups and better software, will come in the next years. Hopefully, those advances will continue addressing the complexity of TB disease.

RNA-Seq and microarrays are yet prohibitive for routine clinical use or even some research projects, because of their costs and expertise required. Commonly, RT-qPCR is used instead at large scale studies to measure a limited signature of candidate genes. This procedure is faster, cheaper, easier to use, and requires minimal electric power. Nonetheless, it is expected that technical progress will end up reducing prize and complexity. Currently, the five platforms most commonly used for RNA-seq include Illumina, SOLiD, Ion Torrent, PacBio and 454 Life sciences, with differences in read length and accuracy. It is likely that, in the near future, more companies will appear in the market, with new reagents and kits available.

The standardization of the process, including preparation of samples and analysis pipelines, would allow a better comparison between laboratories. The goal is to obtain a robust workflow requiring minimal resources to ensure feasibility and data reproducibility amongst the highly variable clinical infrastructures worldwide.

It is also crucial to count on reliable databases. Metadata must be meticulously filled when uploading our results to a public database. They must include a detailed experimental procedure, cellular composition, RNA extraction details and sequencing methods. Better integration among databases will also contribute to easily find gene sets of interest.

There is a growing need of specialists in bioinformatic infrastructures, as Supercomputing Centers, to process and store data. A large combination of bioinformatics software tools and resources is required to process the large volume of raw sequences and the multiple steps in data analysis.

Education in Biocomputing at different levels (university, master, doctorate), will increase the number of persons with expertise in this field. Furthermore, translating big data into simple and useful biological conclusions is usually challenging. The expansion of knowledge in the biological context will help finding interactions among differentially expressed (DE) genes or pathways of biological significance.

One of the most promising approaches to boost the HTTr data utilities is the application of machine learning technology. Machine learning methods benefit from large datasets in order to identify complex relationships between gene candidates. It allows the integration of different datasets into one single analysis, helping to overcome the limitations of typically small sample sizes. Using this approach, one can benefit from all data available in public repositories to learn from large, heterogeneous datasets and build class-prediction models that help on the classification of any sample, regardless of its origin. This is particularly important in TB, where it is of special interest to find a gene combination that could be used for diagnosis in multiple TB-burden settings.

As it has happened with DNA sequencing, emerging technologies will update the current transcriptomic platforms. New technologies that could be more reliable, faster, requiring less amount of sample, and able to detect low abundant transcripts, are expected to come in the next years. As an example, a new generation of sensing technology that uses nanopore, developed by Oxford Nanopore, is suitable for sequencing RNA without conversion to cDNA. Furthermore, gene expression technology can be combined with other platforms, as is the case of spatial transcriptomics. It uses spatially barcoded mRNA-binding oligonucleotides, to obtain a 2D picture of gene expression within a histological section of tissue. Nevertheless, this technology does not allow yet the study of the complete transcriptome.

Integration of information coming from different sources (clinical, immunological) and other omics data (genomic, transcriptomic, epigenomics, proteomic, metabolomics, glycomics, glycoproteomics) will provide an integrative analysis of the factors involved in disease, embryology, evolution or other processes. This field of study, termed systems biology, may improve with the help of new sophisticated computational tools. In recent years, researchers tried to integrate transcriptomic and proteomic data in TB, finding usually a poor correlation. It is expected for systems biology to explain the complex regulatory mechanism that control RNA and protein levels.

**FIGURE 3:**
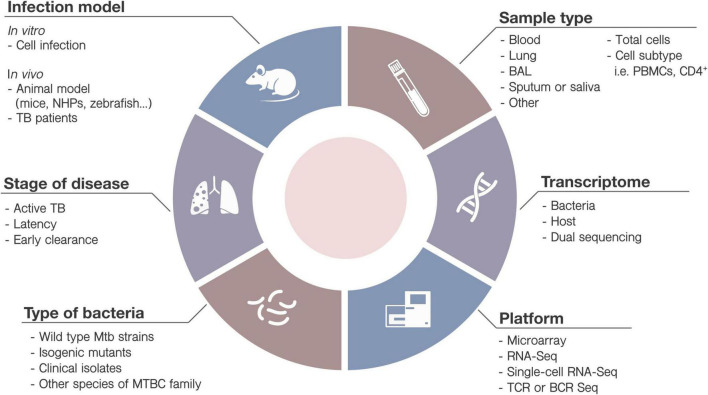
Variables when designing high-throughput transcriptomic studies. Diverse parameters must be taken into account when designing new TB studies involving HTTr. Some of them include: (1) the infection model: *in vitro* (cell infection) or *in vivo* (animals or TB patients) (2) type of bacteria (i.e., strain, mutated or wild-type) and culture conditions; (3) samples to be collected (saliva, sputum, blood, etc.), including analysis of immune cells or subpopulations, at basal level or after stimulation; (4) the stage of the disease (i.e., active disease, latency, early clearance); (5) target element: bacteria, host or both, and (6) the HTTr platform most adequate for the study.

As we have seen along this review, HTTr has proven to be an important tool to reveal interesting biological pathways in TB. Nonetheless, forthcoming studies must take into consideration some critical points:

• Many external factors can affect the transcriptional results. They include the nature of the sample collection (bronchoalveolar lavage, lung parenchyma, saliva, blood), sample processing (whole sample, cell subset isolation, cell subset-depletion) or sequencing platform. Mtb strains, ethnically diverse cohorts and misclassification of patient, will also affect in the transcriptomic signature. Findings in a cohort might not be effectively translated into global clinical use. Researchers must be aware of the variables when interpreting results and designing experiments (Figure 3).

• There must be a partial reliability of the blood transcriptome, as it may not truly represent the processes occurring within the lungs. On the other hand, animal models may not be the best way to study the human TB disease. Some models recreate better the pathology of human TB (guinea pig, macaques) than others (mice). Differences regarding number of exposures, bacterial concentration, challenge route, kinetics, type of lesions produced or illness severity, should also be taken into consideration.
• Further biological studies and clinical validation must be pursued to elucidate the role of the product of DE candidate genes. Moreover, gene expression methods cannot predict the final protein existence, nor their post-translational modifications or evaluate the presence other molecules like polysaccharides, lipopolysaccharides and glycolipids. Complementary studies are required in order to confirm the role of candidate molecules.
• Lastly, special adaptations of the transcriptomic technology are opening new horizons: Single-cell RNA sequencing is a promising technique to explore cell heterogeneity within a population and reveal novel signatures and cell subsets. T cell receptor (TCR) and B cell receptor (BCR) sequencing can use bulk or single-cell methods to characterize complex T and B cell responses, respectively. These techniques allow the identification of all potential clonotypes in a diverse repertoire of TCRs and BCRs, and it is a powerful tool for studying the distribution of lymphocyte repertoires.

## Conclusion

Once accepted the extraordinary plasticity of mechanisms involved in *Mycobacterium tuberculosis*-host interactions, a lot of work remains. HTTr have been immensely valuable in advancing on TB research. It can be applied to a variety of samples and experimental conditions depending on the question asked. Unfortunately, differences in experimental procedures, sequencing platforms, or incomplete metadata usually hamper further exploitation of public-available data.

Currently, RNA-Seq offers key advantages over the rest of existing technologies of HTTr, although it might be replaced in the near future. There is a growing need of bioinformatics specialists and infrastructures that must be met to fully exploit the generated HT data. Lastly, we encourage the creation of interdisciplinary projects that integrates HTTr with the rest of *omic* sciences.

## Search Strategy and Selection Criteria

References were searched through PubMed by use of the terms “Tuberculosis,” “Transcriptomics,” “Microarray,” and “RNA-Sequencing,” for articles published from 1980 to November 2021. Then, articles were ascertain to use high-throughput transcriptomics methods. A selection of most relevant publications was performed based on number of citations, quality and impact of publication journal or relevance in the field.

## Author Contributions

AM-P and OE performed literature search, figures, and writing of the manuscript. ÁG-F made the critical revision of the manuscript and contributed with funding acquisition. All authors contributed to the article and approved the submitted version.

## Conflict of Interest

The authors declare that the research was conducted in the absence of any commercial or financial relationships that could be construed as a potential conflict of interest.

## Publisher’s Note

All claims expressed in this article are solely those of the authors and do not necessarily represent those of their affiliated organizations, or those of the publisher, the editors and the reviewers. Any product that may be evaluated in this article, or claim that may be made by its manufacturer, is not guaranteed or endorsed by the publisher.
